# Evaluation of Cyclooxygenase-2 and p53 Expression in Pterygium Tissue Following Preoperative Intralesional Ranibizumab Injection

**DOI:** 10.3389/fmed.2021.733523

**Published:** 2021-12-24

**Authors:** Ahmad Razif Omar, Mohtar Ibrahim, Hasnan Jaafar, Ab Hamid Siti-Azrin, Embong Zunaina

**Affiliations:** ^1^Department of Ophthalmology and Visual Science, School of Medical Sciences, Universiti Sains Malaysia, Kubang Kerian, Malaysia; ^2^Hospital Universiti Sains Malaysia, Kubang Kerian, Malaysia; ^3^Department of Pathology, School of Medical Sciences, Universiti Sains Malaysia, Kubang Kerian, Malaysia; ^4^Biostatistics and Research Methodology Unit, School of Medical Sciences, Universiti Sains Malaysia, Kubang Kerian, Malaysia

**Keywords:** anti-vascular endothelial growth factor, cyclooxygenase-2, primary pterygium, immunohistochemistry 2, p53 expression

## Abstract

**Introduction:** Overexpression of vascular endothelial growth factor (VEGF), cyclooxygenase-2 (COX-2), and p53 are the postulated aetiopathogenesis in pterygium. VEGF is responsible for the induction of COX-2 expression, whereas p53 plays an important role in the regulation of VEGF. This study aimed to evaluate the immunohistochemistry of COX-2 and p53 expressions from excised pterygium tissue from patients who received intralesional ranibizumab (anti-VEGF) injection 2 weeks prior to pterygium surgery.

**Materials and Methods:** An interventional comparative study involving patients presenting with primary pterygium was conducted between September 2015 and November 2017. The patients were randomized into either the intervention or control group. Patients in the intervention group were injected with intralesional ranibizumab (0.5 mg/0.05 ml) 2 weeks prior to surgery. Both groups underwent pterygium excision followed by conjunctival autograft. Immunohistochemistry staining was performed to evaluate COX-2 and p53 expressions in the excised pterygium tissue.

**Results:** A total of 50 patients (25 in both the intervention and control groups) were recruited. There were 34 (68%) patients with grade III pterygium and 16 (32%) patients with grade IV pterygium. There was statistically significant difference in reduction of COX-2 expression in the epithelial layer [84.0% (95% CI: 63.9, 95.5)] (*p* = 0.007) and stromal layer [84.0% (95% CI: 63.9, 95.5)] (*p* < 0.001) between intervention and control groups. There was no significant difference in the reduction of p53 expression between the two groups.

**Conclusion:** This study demonstrated the possible use of intralesional anti-VEGF treatment prior to pterygium excision as a potential future modality of adjunctive therapy for pterygium surgery.

## Introduction

Pterygium appears as a triangular, wing-like fibrovascular growth from the bulbar conjunctiva over the limbus onto the cornea. Human pterygium exhibits both degenerative and hyperplastic conditions and is characterized by chronic proliferative fibrovascular tissue growth (1–3). Pterygium is a common ocular surface disease in countries in equatorial locations, namely, Malaysia. Its prevalence is as high as 22% in equatorial areas (compared to 2% in latitudes above 40°) (4). However, total prevalence worldwide ranges from 0.7 to 33% (5).

Pterygium has been associated with environmental factors, such as UV radiation, heat, dust, dryness, windy atmospheres, and increasing age (6, 7). Current studies suggested that UV radiation is the most important factor for pterygium formation (3, 8). It is postulated that UV-B radiation causes activation of the nuclear factor-kappa B (NF-kB) signaling pathway and leads to overexpression of cyclooxygenase-2 (COX-2) and vascular endothelial growth factor (VEGF). Overexpression of COX-2 and VEGF plays an important role in the angiogenesis and proliferating of fibrovascular tissue in the formation of pterygium (9, 10). Recent studies have also reported that overexpression of VEGF is produced by corneal fibroblasts in response to local conjunctival inflammation caused by environmental UV radiation, dryness, or dust (11–14). Park et al. (15) found that there was strongly expressed COX-2 and VEGF in macrophages of pterygium tissue. In addition, COX-2 overexpression is correlated with vessel density and VEGF expression (16).

Cyclooxygenase-2 is an enzyme that is responsible for inflammatory cytokine-induced angiogenesis. It is rapidly induced by growth factors, cytokines, and tissue damage. Some studies have reported that COX-2 overexpression was found in more than 80% of pterygium tissues (15, 17). Chiang et al. (17) suggested that COX-2 may play a role in pterygium formation. They reported that 75 out of 90 (83.3%) pterygium specimens were stained positive for COX-2, while all specimens from normal conjunctiva and limbi were stained negative for COX-2. In another study, COX-2 expression was detected in all pterygium tissues, while no COX-2 expression was observed in normal conjunctiva (15). Strong COX-2 expression has also been suggested as one of the risk factors for the recurrence of pterygium (18, 19). In addition to environmental factors, various theories have been postulated for the etiopathogenesis of pterygium, such as expression of p53 oncogenes, genetic and hereditary factors, and infective and immunological factors (3, 20–23).

The gene p53 (wild type) is a tumor suppressor gene that controls the cell cycle (24). Tumor suppressor genes protect cells from converting to cancer cells (25). The p53 gene product, the p53 protein, is a nuclear phosphoprotein, which binds to the DNA (26). In normal cells, wild-type p53 proteins have a short half-life, and the concentration is very low. They are almost undetectable by immunohistochemistry, so the cells stain negative (24, 26, 27). Mutations in the p53 gene can lead to the synthesis of abnormal p53 proteins, which has increased in protein stability resulting in prolonged half-life (26, 27). In many types of neoplastic cells, its concentration is higher hence immunohistochemical staining for the p53 protein becomes positive (24, 26). p53 expression is an indicator of p53 mutation (27, 28).

Ultraviolet radiation can inactivate p53 through mutation and subsequently lead to cell proliferation and genomic instability (29). Overexpression of p53 was found in the epithelium of pterygium tissue, thus suggesting that pterygium could be a result of uncontrolled cell proliferation (26). Expression of p53 in primary pterygium varies between 54% and 100% (26, 30–32). Kieser et al. (33) reported that p53 mutation is associated with VEGF expression.

Ranibizumab (Lucentis® Genentech, Inc., South San Francisco, CA, United States) is a recombinant humanized anti-VEGF antibody that binds to VEGF-A. Through binding to VEGF-A, ranibizumab interrupts the VEGF-receptors interaction and thus prevents new vessels proliferation. Several studies (9, 34, 35) have demonstrated the beneficial effects of subconjunctival anti-VEGF, not only in regression of pterygium size and proliferation of vessels but also in the reduction of recurrence rate. However, there has been no study done to evaluate the immunohistochemistry of COX-2 and p53 expressions following anti-VEGF administration.

Therefore, the aim of this study was to compare the immunohistochemistry of COX-2 and p53 expressions from excised pterygium tissues following intralesional ranibizumab injection 2 weeks prior to pterygium surgery vs. the control group. Intralesional anti-VEGF treatment may provide future modalities of adjunctive therapy for pterygium surgery.

## Materials and Methods

An interventional comparative study involving patients with primary pterygium was conducted between September 2015 and November 2017. The study followed the tenets of the Declaration of Helsinki and was approved by the local ethical boards USM/JEPeM/[269.3 (1)].

The sample size was calculated using PS Power and Sample Size Calculation 3.1.2 based on an independent, prospective, two-proportion, and uncorrected chi-square test design. Power was set at 0.8, and the input was as follows: α = 0.05, p0 = 0.7, p1 = 0.3, m = 1. The estimated sample size was based on several studies showing that COX-2 expression in primary pterygium is 80–100% (15, 17, 19). The calculated sample size was 25 with a 10% dropout. A total of 50 patients with primary pterygium were recruited for this study (25 patients for the intervention group and 25 patients for the control group).

This study was conducted among patients with grades III–IV primary nasal pterygium. Those patients with temporal or double-headed (nasal and temporal) pterygium, previous ocular surgery or trauma, ocular surface disorder, corneal pathology, primary or secondary glaucoma, history of taking regular eye drops, or history of a systemic thromboembolic event were excluded from this study. Pregnant women and lactating mothers were also excluded.

Patients who fulfilled the selection criteria and agreed to undergo surgical excision of pterygium were explained the nature of the study, and written consent was obtained. Data regarding demographics (age and gender), medical history, previous ocular surgery or treatment, and previous systemic diseases were obtained through direct questioning from patients and from medical records.

### Clinical Classification of Pterygium

A thorough slit lamp examination was performed by an independent ophthalmologist to clinically grade the pterygium. The clinical classification of pterygium was based on a modified classification system (36). The pterygium was rated as stage I (tissue involvement of the limbus), stage II (the tissue just on the limbus), stage III (the tissue between the limbus and pupillary margin), and stage IV (tissue central to the pupillary margin).

### Randomization

Patients with grades III–IV primary nasal pterygium only were selected. Those who agreed to participate in the study were randomized into either the intervention or control group. The eligible patients were randomized into two groups by using the opaque sealed envelope technique. A piece of paper written with “Intervention” was placed in 25 envelopes, and the remaining 25 envelopes were written with “Control”. These envelopes were shuffled and stored at the randomization room after random numbering the allocation sequence that was generated using a digital table by the primary investigator. The envelope was drawn for each patient by a co-investigator, who was not involved in the preparation of these opaque sealed envelopes. Patients in the intervention group were given an intralesional injection of ranibizumab. Patients in the control group were not given any injection or placebo.

### Intralesional Injection of Ranibizumab

Patients in the intervention group were given an intralesional injection of ranibizumab (Lucentis® Genentech, Inc., United States) 2 weeks before their pterygium surgery. The injection was given in an operating theater using an aseptic technique. The patient was lying supine, and the eye was anesthetized with topical proparacaine (0.5%). Then, the eye was cleaned and draped, and a lid speculum was applied. Topical povidone (5%) was applied to the conjunctiva and then washed thoroughly. Ranibizumab 0.5 mg/0.05 ml was then injected into the body of the pterygium along the limbus. After the procedure, each patient was treated with 0.5% topical chloramphenicol every 6 h for 1 week. Each patient was scheduled for pterygium excision surgery 2 weeks post-procedure. This 2-week post-intralesional ranibizumab period was chosen for pterygium excision because a study done by Mandalos et al. (37) showed that pterygium attained the lowest vessel density after 2 weeks of anti-VEGF injection.

### Pterygium Surgery

Patients in both the intervention and control groups were admitted for the pterygium surgery procedure. General and ocular assessments had been performed preoperatively. Any side effects or complications related to intralesional injection of ranibizumab were recorded for those in the intervention group.

The pterygium excision surgery was carried out in an operating theater under topical anesthesia using 5% proparacaine. Topical phenylephrine (2.5%) was given to reduce bleeding during the excision. The surgical area was then cleaned and draped, and a lid speculum was applied. Topical povidone (5%) was applied to the cul-de-sac for 3 min and then washed thoroughly. Intralesional lignocaine (1%) was injected into the pterygium body. The pterygium was excised from the apex to the base using a Tooke corneal knife and Westcott scissors. Part of the pterygium head was excised, and fibrovascular tissue was scraped using a Tooke corneal knife. Hemostasis was achieved by applying pressure to the bleeding site using cotton bud tips. Conjunctiva autograft was harvested from the superior or inferior bulbar conjunctiva and then applied with the correct orientation of limbal edge to the bare sclera. The conjunctival autograft was either secured using fibrin adhesive glue (Tisseel VH; Baxter Healthcare Corp., Deerfield, IL, USA) or sutured using absorbable suture vicryl 8/0 depending on the availability of fibrin adhesive glue during the surgery.

This part of this study was open-label in which both the patient and the operating surgeon were aware of the treatment allocation.

### Paraffin-Embedded Tissue Block Preparation

Excised pterygium tissue was transported to the pathology laboratory in 10% normal buffered formalin for tissue fixation for histological diagnosis. After 48 h, the formalin was exchanged with 80–95% ethanol and absolute alcohol for the tissue dehydration procedure, and the excised pterygium tissues were processed and embedded in paraffin wax. The tissue processing and paraffin-embedded tissue blocks had been prepared by the principal investigator, co-investigator, and laboratory technician.

### Immunohistochemical Analysis of COX-2

Sections of approximately 3–4 μm in thickness were obtained from paraffin-embedded tissue blocks. All sections were deparaffinized in xylene, rehydrated through a graded series of alcohols, and washed with phosphate-buffered saline. This buffer was used for all subsequent washes. Sections for COX-2 detection were heated in a pressure cooker for 3 min at full pressure in citrate buffer (pH 6.0). Dako REAL Peroxidase blocking solution (Dako, Denmark) was used to block endogenous peroxidase and was incubated for 5 min. Monoclonal mouse anti-COX-2 antibody at a dilution of 1:200 (Clone CX-294; Dako, North America) was used as the primary antibody. The primary antibody was incubated with the tissue sample to allow binding to the target antigen. The incubation time was 1 h at room temperature. Dako REAL EnVision Horse Reddish Peroxidase (HRP) rabbit/mouse (ready-to-use) (Dako, Denmark) was the secondary antibody, with specificity for the primary antibody, was incubated with the tissue sample to allow binding to the primary antibody. This incubation step was 30 min at room temperature. Signals were developed with 3,3'-diaminobenzidine (DAB) for 5 min and counterstained with hematoxylin. Negative controls were obtained by leaving out the primary antibody. COX-2 expression in colon adenocarcinoma tissue was used as a positive control.

Clone CX-294 (Clone CX294; Dako North America) is specific to the peptide used for immunization. In Western blotting and immunoprecipitation experiments, CX-294 was shown to identify the inducible human COX-2 using interleukin-1 alpha-stimulated human umbilical vein endothelial cells (HUVEC); peptide blocking of the antibody with the COX-2 immunogen eliminated all reactivity.

Evaluation and scoring of immunohistochemical staining of COX-2 expression were performed by two independent researchers for test-retest reliability. Both researchers were single blinded to the treatment allocation. COX-2 expression in the epithelial layer was scored according to the percentage of positive cell staining: score 0, negative staining; score 1+, 1–10% positive cell staining; score 2+, 11–50% positive cell staining; and score 3+, >50% positive cell staining. COX-2 expression in the stromal layer was scored according to the average number of COX-2-expressing cells calculated over ten random high-power fields (magnification 400×): score 0, negative staining; score 1+, 1–5 positive cells staining; score 2+, 6–10 positive cells staining; and score 3+, >10 positive cells staining. The scoring method that was used in this study was based on the scoring described by Park et al. (15).

### Immunohistochemical Analysis of p53

Sections of approximately 3–4 μm in thickness were obtained from paraffin-embedded tissue blocks. All sections were deparaffinized in xylene, rehydrated through a graded series of alcohols, and washed with phosphate-buffered saline. This buffer was used for all subsequent washes. Sections for p53 detection were heated in a pressure cooker for 3 min at full pressure in crisEDTA buffer (pH 9.0). Dako REAL Peroxidase blocking solution (Dako, Denmark) was used to block endogenous peroxidase and was incubated for 5 min. Monoclonal mouse anti-p53 antibody, DO-7, at dilution 1:100 (Code number M 7001; Dako, Denmark) was used as the primary antibody. The primary antibody was incubated with the tissue sample to allow binding to the target antigen. The incubation time was 1 h at room temperature. Dako REAL EnVision (HRP rabbit/mouse (ready-to-use) (Dako, Denmark) was the secondary antibody, with specificity for the primary antibody, was incubated with the tissue sample to allow binding to the primary antibody. This incubation step was 30 min at room temperature. Signals were developed with DAB for 5 min and counterstained with hematoxylin. Negative controls were obtained by leaving out the primary antibody. p53 expression in colon adenocarcinoma tissue was used as a positive control.

Evaluation and scoring of immunohistochemical staining of p53 expression were performed by two independent researchers for test-retest reliability. Both researchers were single blinded to treatment allocation. p53 expression was observed in the nuclei of the epithelial layer of excised pterygium tissue. The immunohistochemical p53 results were scored according to the percentage of cells with positive nuclei staining: score 0, negative staining in the nuclei; score 1+, 1–10% positive staining in the nuclei; score 2+, 11–50% positive staining in the nuclei; score 3+, >50% positive staining in the nuclei. The scoring method that was used in this study was based on the scoring described by Tsai et al. (38).

### Statistical Analysis

Statistical analysis was carried out using the Statistical Package for Social Sciences (SPSS) version 22. Fisher's exact test was used for the association of COX-2 and p53 expressions between the intralesional ranibizumab and control groups. Fisher's exact tests were conducted using STATA version 14, and a value of *p* <0.05 indicated statistical significance.

## Results

### Demographic Data

A total of 50 patients (25 patients in the intervention group and 25 patients in the control group) were recruited. Among these two groups, there were 34 (68%) patients with grade III pterygium and 16 (32%) patients with grade IV pterygium. There were 13 (52%) men and 12 (48%) women in each group. Ages in the intervention group ranged from 36 to 67 years, with a mean age of 52.64 ± 8.8 years. In the control group, ages ranged from 37 to 68, with a mean age of 51.96 ± 9.5 years (Table 1).

**Table 1:** Distribution of age, gender, and method of conjunctival autograft for both groups.

**Parameters**	**Intervention**	**Control**
	***n*** **= 25**	***n*** **= 25**
**Age (years)**		
Mean	52.64 ± 8.8	51.96 ± 9.5
Range	36–67	37–68
**Gender (n, %)**		
Male	13 (52)	13 (52)
Female	12 (48)	12 (48)
**Methods of conjunctival autograft (n, %)**		
Fibrin glue	18 (72)	22 (88)
Suturing	7 (28)	3 (12)

Post-pterygium excision, the conjunctival autograft was performed using fibrin adhesive glue in 18 (72%) patients in the intervention group and 22 (88%) patients in the control group. Suturing of conjunctival autograph was done in the other 10 patients (seven patients in intervention and three patients in control groups) (Table 1). There were no side effects or complications post-intralesional ranibizumab injection observed among the patients in the intervention group.

### COX-2 Expression in Excised Pterygium Tissue

Cyclooxygenase-2 expression was observed in both the epithelial layer and stromal layer of excised pterygium tissue. There were a greater number of positive cells staining in the epithelial layer as compared to the stromal layer. The epithelial layer showed few focal areas of hyperplasia indicate a disturbance of cell proliferation. Immunohistochemical scoring of COX-2 expression of pterygium tissues showed different staining intensities from weak to moderate and strong staining intensity.

In the epithelial layer, COX-2 expression was observed mainly in the basal epithelia. The distribution of COX-2 expression in epithelial cells between the two groups is shown in Table 2. Higher scoring (score 3+) was seen more in the control group as compared to the intervention group. There were 4 (16%) pterygium tissues in the intervention group that showed negative epithelial cell staining of COX-2. None of the tissues from the control group showed negative staining of COX-2 in epithelial cells. There was a statistically significant difference in the reduction of COX-2 expression in the surface epithelium between intervention and control groups [84.0% (95% CI: 63.9, 95.5)] (*p* = 0.007) post-intralesional injection of ranibizumab.

**Table 2:** Association of cyclooxygenase-2 (COX-2) and p53 expression in excised pterygium tissue between intervention and control groups post-intralesional injection of ranibizumab.

**Immunohistochemical analysis**	**Intervention group** ***n*** **(%)**	**Control group** ***n*** **(%)**	**Point estimate**	**95% CI**	***p*** **value**
**COX-2 epithelial cells staining**					
Score 0	4 (16)	0 (0)			
Score 1+	10 (40)	6 (24)	84.0%	63.9, 95.5	0.007
Score 2+	10 (40)	10 (40)			
Score 3+	1 (4)	9 (36)			
**COX-2 stromal cells staining**					
Score 0	4 (16)	0 (0)			
Score 1+	15 (60)	6 (24)	84.0%	63.9, 95.5	<0.001
Score 2+	6 (24)	14 (56)			
Score 3+	0 (0)	5 (20)			
**p53 epithelial cells staining**					
Score 0	1 (4)	0 (0)			
Score 1+	9 (36)	3 (12)	96.0%	79.6, 99.9	0.085
Score 2+	9 (36)	10 (40)			
Score 3+	6 (24)	12 (48)			

*Fisher's exact test, p < 0.05 significant*.

*CI, confidence interval; COX-2, cyclooxygenase-2*.

In the stromal layer, COX-2 staining was observed in stromal inflammatory cells, capillary vessels, and fibroblasts. The distribution of COX-2 expression in stromal cells between the two groups is shown in Table 2. Score 2+ was observed higher in the control group as compared to the intervention group. On the contrary, a score 1+ was higher in the intervention group as compared to the control group. There were 4 (16%) pterygium tissues in the intervention group that showed negative stromal cell staining of COX-2. None of the tissues from the control group showed negative staining of COX-2 in stromal cells. There was a statistically significant difference in the reduction of COX-2 expression in stromal cells between intervention and control groups [84.0% (95% CI: 63.9, 95.5)] (*p* < 0.001) post-intralesional injection of ranibizumab.

### p53 Expression in Excised Pterygium Tissue

p53 immunostaining was observed in the nuclei of the surface epithelia. The distribution of p53 expression in epithelial cells between the two groups is shown in Table 2. Intervention group showed 4% negative staining, 36% in score 1+, and 24% in score 3+ as compared to control group with 0, 12, and 48%, respectively. There was only one (4%) pterygium tissue in the intralesional ranibizumab group that showed negative epithelial cell staining of p53, and none from the control group. However, there was no significant difference in the reduction of p53 expression between intervention and control groups [96.0% (95% CI: 79.6, 99.9)] (*p* = 0.085) post-intralesional injection of ranibizumab.

Immunohistochemical scoring of COX-2 expression in the epithelial layer, COX-2 expression in the stromal layer, and p53 expression in the epithelia layer of pterygium tissues are shown in Supplementary Figures 1–3, respectively, as supplementary files. In view of the inability to use of scale slide, the images were captured without a scale bar.

## Discussion

There are various proposed etio-histopathologies of pterygia. Overexpression of VEGF in response to numerous stimuli plays a role in pterygium formation (11–14). Apart from that, several studies have shown that COX-2 and p53 overexpressions play important roles in the pathogenesis of pterygium. COX-2 is the significant enzyme for inflammatory cytokine-induced angiogenesis, and it was found in 80–100% of primary pterygia (15, 17, 19). Expression of p53 in primary pterygia varies between 54 and 100% (26, 30–32). It has been suggested that pterygium could result from uncontrolled cell proliferation (26).

Several studies have demonstrated the effectiveness of subconjunctival anti-VEGF in regression of pterygium size (9), vascularity (9, 39, 40), and the recurrence rate (39, 41). VEGF is a regulator of angiogenesis and inhibition of VEGF can prevent new vessel proliferation (9). Mohamed et al. (40) demonstrated that pterygium with anti-VEGF injection showed a reduction in mean vessel count and VEGF expression.

Vascular endothelial growth factor is induced by hypoxic stimuli and is responsible for the induction of COX-2 expression in endothelial cells (42). In our study, there were significant reductions in COX-2 expression in the surface epithelium (*p* = 0.007) and stromal cells (*p* < 0.001) in pterygium tissue post-intralesional injection of ranibizumab as compared to the control group. Liu et al. (10) reported that there was a significant correlation between COX-2 upregulation and VEGF expression in pterygium tissue. They found that the expression of COX-2 showed a significant correlation with the density of the microvessels. The finding was supported by Mohamed et al. (40) showed that intralesional injection of anti-VEGF in pterygium decreased vascularity and VEGF expression. We postulated that inhibition of VEGF is associated with a reduction of COX-2 expression and vascularity.

However, only 16% of pterygium tissue in the intervention group in our study showed negative staining for COX-2 expression. Multiple factors might be responsible for COX-2 expression. Application of COX inhibitor demonstrated inhibition of COX-2 expression (43), indicating COX-2-prostaglandin-E2 pathway is the possible mechanism (44).

Abnormal expression of p53 has also been demonstrated in pterygium suggesting that p53 plays a possible role in the pathogenesis of pterygium (28). The expression of p53 could be associated with an increase in VEGF production (45). In our study, p53 immunostaining was observed in the nuclei of the surface epithelium. Although there was no significant difference in the reduction of p53 expression in pterygium tissue post-intralesional injection of ranibizumab between the two groups (*p* = 0.085), the trend of scoring is more favorable in the intervention group. Lower scoring (score 1+) was seen more in the intervention group as compared to the control group. Our finding showed that VEGF inhibition plays a minor role in the reduction of p53 expression. We postulated that the possibility of UV radiation-induced p53 mutation is the major mechanism for p53 expression.

Several studies have reported that there is a relationship between UV radiation and the prevalence of pterygium (46–48). Those who are living at high altitudes (above 3,000 m) were exposed to high UV-B sunlight and had high prevalence rates of pterygium (46, 47) and those who are living at low altitudes (below 3,000 m) had a low prevalence of pterygium (48). Recent reports have suggested that p53 expression in the epithelium of pterygium is probably a result of UV radiation exposure (24, 26, 30, 32). p53 expression was observed to be increasing with increasing duration and severity of pterygium (49).

The different scoring system of COX-2 expression was used for the epithelial layer and stromal layer. Scoring according to the percentage of positive cell staining was used in the epithelial layer, whereas in the stromal layer was scored according to the average number of COX-2-expressing cells. Histologically, pterygia are characterized by a hyperplastic, epithelial mesenchymal transition, clusters of basal cells aggregation beside an activated fibroblastic stroma with inflammation and matrix remodeling (1). Small clusters of aggregation basal cells illustrated smaller cell size and had increased nuclear-to-cytoplasm ratio (1). Dense epithelial cells in a few clusters of epithelial layers with different staining intensity, COX-2 expression in the epithelial layer was scored according to the percentage of positive cell staining. On the other hand, absolute cell number was used for scoring of cell staining in the stromal layer in view of less abundant stromal cells.

With the emergence and availability of anti-VEGF, intralesional of anti-VEGF on pterygium tissue was suggested as a possible adjunctive therapy for pterygium excision by decreasing the blood vessel formation (9, 40, 50). Strong COX-2 expression has been suggested as a risk factor for the recurrence of pterygium (18, 19). However, p53 expression in the epithelium overlying the pterygium is not associated with the recurrence of pterygium (51).

The use of subconjunctival anti-VEGF injection as an adjunct therapy to the surgery is relatively safe (40, 52). There were no side effects or complications post-intralesional ranibizumab injection observed in our study.

### Limitation and Recommendation

This study does have some limitations. Firstly, the specific mechanism of COX-2 and p53 overexpressions was not fully understood. Therefore, detailed information about each patient's lifestyle, sun exposure, irradiation, family history, and medication intake should be obtained and considered during analysis, as all these factors can affect COX-2 and p53 overexpressions in pterygium tissue.

In this study, the sealed envelope technique was used as the technique for the randomization method. However, this sealed envelopes technique is not a robust method for allocation concealment. Other methods of randomization such as block or stratified randomization are the better randomization technique in order to produce a comparable group and eliminate the source of bias in the intervention group.

The COX-2 expression in the stromal layer was analyzed as a total of all cells. Analysis of the COX-2 expression according to the different stroma cell types was not performed. The information regarding COX-2 expression according to the different stroma cell types might strengthen the findings. We recommend analyzing the COX-2 expression based on the different types of cells in the stromal layer in future studies.

Images of immunohistochemical scoring of COX-2 expression and p53 expression in pterygium tissues (Supplementary Files) were captured without a scale bar because we were not being able to use a scale slide during capturing the image. Since scale bar information allows a quicker and reliable way to assess the size of features shown in images, we advise any microscopic images should have a scar bar either using a scale slide or specific software.

Intralesional ranibizumab would have an effect on VEGF expression and reflect the effect of ranibizumab on the treatment efficacy. However, due to limited funds, VEGF expression was not analyzed in this study. We strongly recommend analyzing the VEGF expression in future studies that used anti-VEGF as a treatment option.

Due to the various postulated etiopathogenesis of pterygium that exists currently, evaluating the effect of intralesional ranibizumab injection on oxidative stress markers in pterygium such as 8-hydroxy-20-deoxyguanosine and on CD34 expression in endothelial cells might be useful.

Lignocaine, is an amide local anesthetic, was administered during the procedure of pterygium surgery. The mechanism of action of lignocaine for local anesthesia is by reversible blockade of nerve fiber impulse propagation (53). It was reported that lignocaine significantly inhibits the VEGF by suppressing VEGF receptor-2 signaling (54, 55). Suppression of VEGF in the intervention group might also be caused by lignocaine administration besides intralesional injection of anti-VEGF. Further study needs to be evaluated to determine the impact of administration of local anesthesia on the effect of COX-2 and p53 overexpression. In our study, both groups were given lignocaine anesthesia during pterygium surgery. Therefore, the effect of VEGF inhibition by lignocaine was standardized in both groups.

In our study, the recurrence rate was not evaluated in both groups. The surgical technique for conjunctiva autograft application post-pterygium excision for our patients was not standardized due to the limited availability of fibrin adhesive glue during the surgeries. In our study, pterygium excision was performed with a conjunctiva autograft application, using either fibrin adhesive glue or absorbable sutures. In view of different surgical techniques, so this will affect the long-term recurrence rates between these two groups. Future studies should standardize the surgical method and evaluate the recurrence rate of pterygium between intervention and control groups.

## Conclusion

Intralesional ranibizumab has the effect of reducing both COX-2 and p53 expressions in primary pterygium tissue compared to the control group. However, only the COX-2 expression showed a significant result. This study demonstrated the possible use of intralesional anti-VEGF treatment prior to pterygium excision as a potential future modality of adjunctive therapy for pterygium surgery.

## Data Availability Statement

The raw data supporting the conclusions of this article will be made available by the authors, without undue reservation.

## Ethics Statement

The studies involving human participants were reviewed and approved by Local Ethical Boards USM/JEPeM/[269.3 (1)] from Universiti Sains Malaysia. The patients/participants provided their written informed consent to participate in this study.

## Author Contributions

AO, MI, and HJ contributed to the design of the study. AO, MI, and HJ were involved in data collection. HJ and AO contributed to laboratory analysis. AS-A and AO contributed to statistical analysis. AO and MI drafted the manuscript. MI and EZ crucially revised the manuscript. All authors have read and approved the final manuscript.

## Funding

This work was supported by a short term grant (Account Number: 304/PPSP/61313145) from Universiti Sains Malaysia.

## Conflict of Interest

The authors declare that the research was conducted in the absence of any commercial or financial relationships that could be construed as a potential conflict of interest.

## Publisher's Note

All claims expressed in this article are solely those of the authors and do not necessarily represent those of their affiliated organizations, or those of the publisher, the editors and the reviewers. Any product that may be evaluated in this article, or claim that may be made by its manufacturer, is not guaranteed or endorsed by the publisher.

## Supplementary Material

Supplementary Figure 1 Immunohistochemical analysis of cyclooxygenase-2 (COX-2) protein expression in the epithelial layer of excised pterygium tissue. **(A)** This panel shows negative expression (no positive cell staining). **(B)** Positive immunostaining of scores 1+, 1–10% positive cells staining. **(C)** Positive immunostaining of scores 2+, 11–50% positive cells staining. **(D)** Positive immunostaining of scores 3+, more than 50% positive cells staining (IHC stain: magnification 200×). IHC, Immunohistochemistry.

Supplementary Figure 2 Immunohistochemical analysis of cyclooxygenase-2 (COX-2) protein expression in the stromal layer of excised pterygium tissue. **(E)** This panel shows negative expression (no positive cell staining). **(F)** Positive immunostaining of scores 1+, 1–5 positive cells staining. **(G)** Positive immunostaining of scores 2+, 6–10 positive cells staining. **(H)** Positive immunostaining of scores 3+, more than 10 positive cells staining (IHC stain: magnification 200×). IHC, Immunohistochemistry.

Supplementary Figure 3 Immunohistochemical analysis of p53 protein expression in the epithelial layer of excised pterygium tissue. **(J)** This panel shows negative expression (no positive staining in the nuclei of all cells). **(K)** Positive immunostaining of scores 1+, 1–10% positive staining in the nuclei. **(L)** Positive immunostaining of scores 2+, 10–50% positive staining in the nuclei. **(M)** Positive immunostaining of scores 3+, more than 50% positive staining in the nuclei (IHC stain: magnification 200×). IHC, Immunohistochemistry.
