# Supplementary figures and images for: Evaluation of Cyclooxygenase-2 and p53 Expression in Pterygium Tissue Following Preoperative Intralesional Ranibizumab Injection
Source: Front Med (Lausanne). 2021 Dec 24;8:733523. doi: 10.3389/fmed.2021.733523 (PMC8739785; doi:10.3389/fmed.2021.733523)

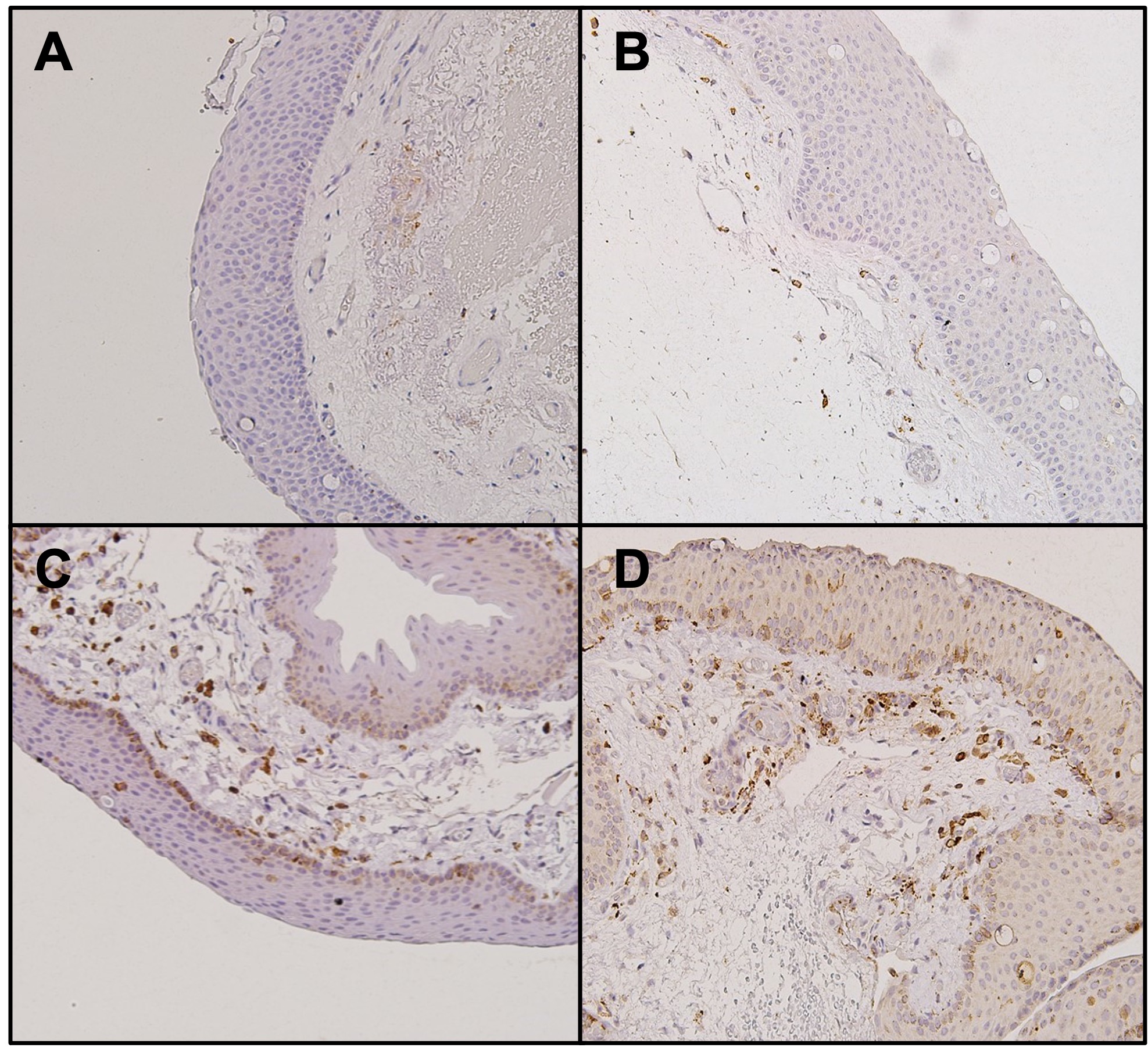

Supplement: Supplementary Figure 1 — Immunohistochemical analysis of cyclooxygenase-2 (COX-2) protein expression in the epithelial layer of excised pterygium tissue. (A) This panel shows negative expression (no positive cell staining). (B) Positive immunostaining of scores 1+, 1–10% positive cells staining. (C) Positive immunostaining of scores 2+, 11–50% positive cells staining. (D) Positive immunostaining of scores 3+, more than 50% positive cells staining (IHC stain: magnification 200×). IHC, Immunohistochemistry. [file Image_1.JPEG]

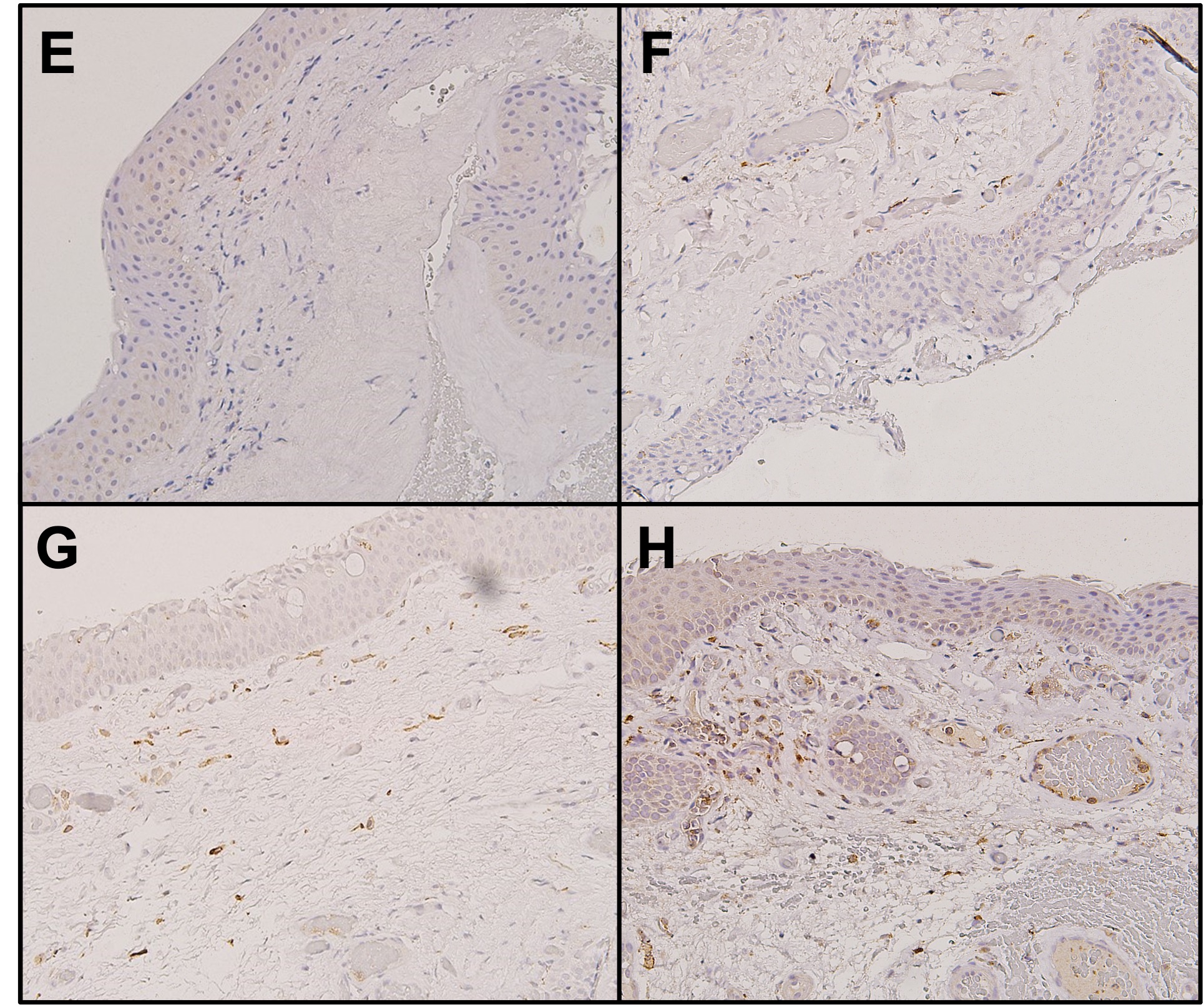

Supplement: Supplementary Figure 2 — Immunohistochemical analysis of cyclooxygenase-2 (COX-2) protein expression in the stromal layer of excised pterygium tissue. (E) This panel shows negative expression (no positive cell staining). (F) Positive immunostaining of scores 1+, 1–5 positive cells staining. (G) Positive immunostaining of scores 2+, 6–10 positive cells staining. (H) Positive immunostaining of scores 3+, more than 10 positive cells staining (IHC stain: magnification 200×). IHC, Immunohistochemistry. [file Image_2.JPEG]

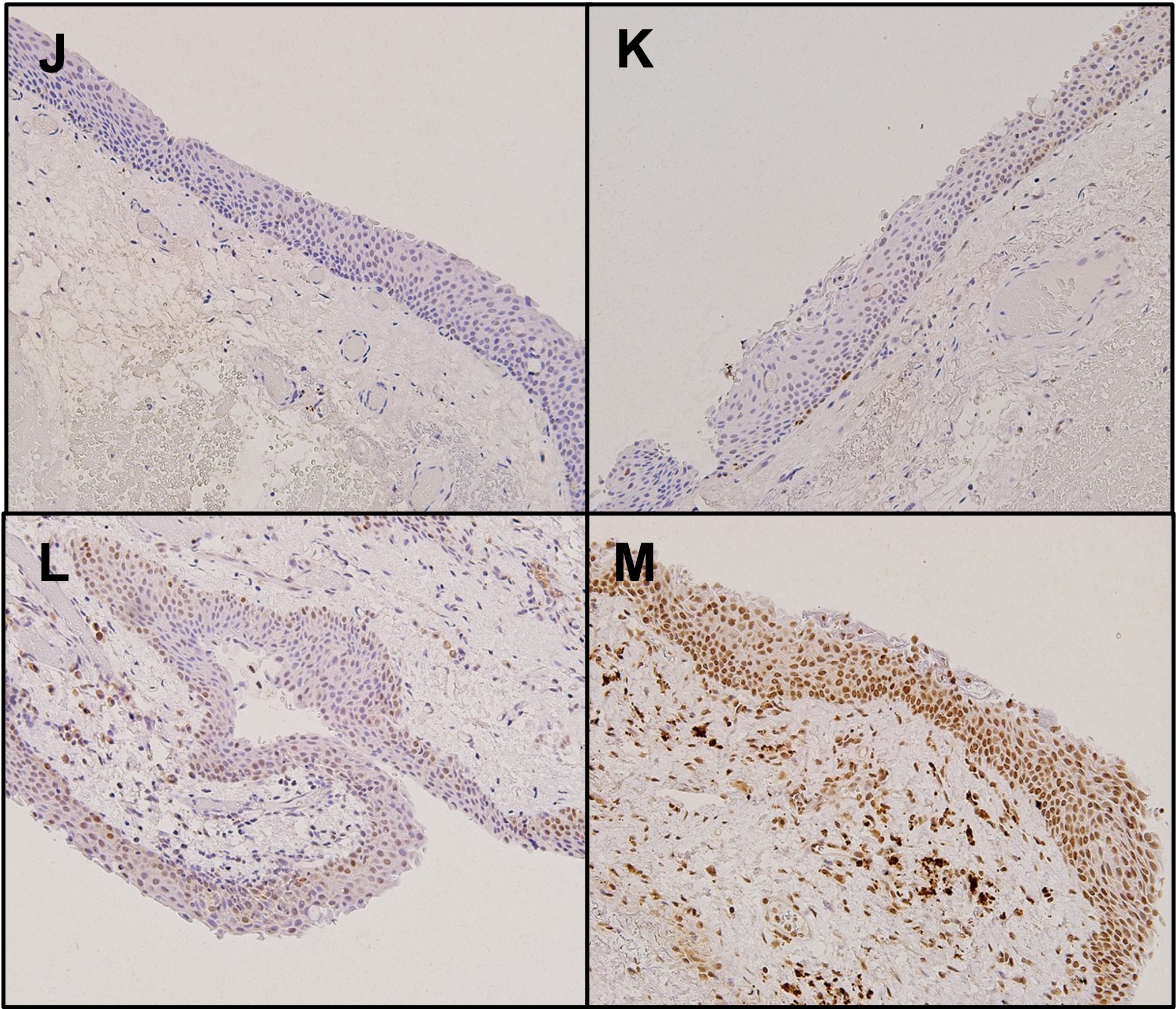

Supplement: Supplementary Figure 3 — Immunohistochemical analysis of p53 protein expression in the epithelial layer of excised pterygium tissue. (J) This panel shows negative expression (no positive staining in the nuclei of all cells). (K) Positive immunostaining of scores 1+, 1–10% positive staining in the nuclei. (L) Positive immunostaining of scores 2+, 10–50% positive staining in the nuclei. (M) Positive immunostaining of scores 3+, more than 50% positive staining in the nuclei (IHC stain: magnification 200×). IHC, Immunohistochemistry. [file Image_3.JPEG]
